# Forced swim stressor: Trends in usage and mechanistic consideration

**DOI:** 10.1111/ejn.15139

**Published:** 2021-03-08

**Authors:** Marc L. Molendijk, E. Ronald de Kloet

**Affiliations:** ^1^ Institute of Psychology Leiden University Leiden The Netherlands; ^2^ Leiden Institute for Brain and Cognition Leiden University Medical Center Leiden The Netherlands; ^3^ Division of Endocrinology Department of Medicine Leiden University Medical Center Leiden The Netherlands

**Keywords:** animal model, brain, forced swim test, glucocorticoid, stress

## Abstract

The acquired immobility response during the “forced swim test (FST)” is not a rodent model of depression, but the test has some validity in predicting a compound's antidepressant potential. Nevertheless, 60% of the about 600 papers that were published annually the past 2 years label the rodent's immobility response as depression‐like behaviour, but the relative contribution per country is changing. When the Editors‐in‐Chief of 5 journals publishing most FST papers were asked for their point of view on labelling immobility as depression‐like behaviour and despair, they responded that they primarily rely on the reviewers regarding scientific merit of the submission. One Editor informs authors of the recent NIMH notice (https://grants.nih.gov/grants/guide/notice‐files/NOT‐MH‐19‐053.html) which encourages investigators to use animal models “for” addressing neurobiological questions rather than as model “of” specific mental disorders. The neurobiological questions raised by use of the FST fall in two categories. First, research on the role of endocrine and metabolic factors, with roots in the 1980s, and with focus on the *bottom‐up* action of glucocorticoids on circuits processing salient information, executive control and memory consolidation. Second, recent findings using novel technological and computational advances that have allowed great progress in charting *top‐down* control in the switch from active to passive coping with the inescapable stressor executed by neuronal ensembles of the medial prefrontal cortex via the peri‐aquaductal grey. It is expected that combining neural *top‐down* and endocrine *bottom‐up* approaches will provide new insights in the role of stress‐coping and adaptation in pathogenesis of mental disorders.

## Graphical abstract

Currently, about 600 papers per year are published that report the use of the forced swim test (FST). The relative contribution to the FST literature per country is changing over the years, as is the interpretation of the behaviour that is observed in this test.

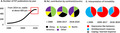

**Abbreviations**
ADX: adrenalectomized
av: anteroventral
BNST: bed nucleus of the stria terminalis
dl: dorsolateral
DSL: dorsolateral striatal
EPM: Elevated Plus Maze
FST: forced swim test
GABA: γ‐aminobutyric acid
HPA: hypothalamic–pituitary–adrenal
LAL: long acttack latency
LDB: Light‐Dark Box
mPFC: medial prefrontal cortex
OFT: Open Field Test
PAG: periaqueductal gray
PVN: paraventricular nucleus
RLA: Roman low avoidance
SAL: short attack latency
SPT: sucrose preference test
TST: tail suspension test
vl‐PAG: ventro‐lateral periaqueductal gray
VTA: ventral tegmental area

**Bruce S. McEwen (1939–2020)**

“What it takes to be a good scientist is persistence, to develop your own story, sticking to it and being a good citizen, a collaborator, because most things you can't do on your own.”

https://www.rockefeller.edu/about/history/oral‐history‐project/interview‐bruce‐mcewen/

With the death of Bruce S. McEwen, January 2, 2020, we lost one of the pioneers in the Neuroscience of Stress. Bruce discovered the receptor sites of corticosterone in the hippocampus of the rat and this discovery in 1968 was one of foundations of the new discipline of Psychoneuroendocrinology (McEwen et al., 1968). The more than 1,000 scientific articles carrying his name are a source of inspiration for students exploring the neurobiology of stress and stress‐related mental disorders. See for in memoriam a.o. (Hill et al., 2020; Lupien & de Kloet, 2020).

## 1 INTRODUCTION

The forced swim test (FST) was developed more than 40 years ago by Roger Porsolt and colleagues as a relatively rapid behavioural screening assay to identify in rats new compounds with a potential antidepressant activity (Porsolt et al., ,^1977^, ^1978^). However, some 20 years later the very same test was promoted as animal model of depression (Cryan & Mombereau, 2004; Dalvi & Lucki, 1999; Porsolt, 2000) and this caused in retrospect an avalanche of FST publications. In 2015 we wrote a commentary to highlight that FST immobility lacked validity as a measure of depression‐like behaviour (Molendijk & de Kloet, 2015). In that commentary, we showed that the usage of the FST to phenotype behaviour of rats and mice in terms of depression‐like and despair increased over time at the expense of labelling immobility or motor inactivity as a passive coping style. The latter interpretation referred to acquired immobility as the default survival mode by saving energy resources (Hawkins et al., 1978).

In 2018, we again examined the usage of the FST and found that the popularity of the test was still increasing and the majority of the authors discussed, without comment, the immobility response as depression‐like, or as despair or helplessness (Molendijk & de Kloet, 2019). The FST was not only used as model of depression; interpretations of immobility as anxiety, psychomotor retardation or autism were also encountered (Anyan & Amir, 2018; Commons et al., 2017; Unal & Canbeyli, 2019). Then we sent a questionnaire to the first or senior authors of the original 84 FST articles who cited our 2015 and 2016 publications on the use and interpretation of the FST (de Kloet & Molendijk, 2016; Molendijk & de Kloet, 2015). Fifty‐two percent of the authors that were contacted, did return the poll and indicated they had changed their qualification of the rodent's FST performance from, for example, depression‐like into passive coping. The respondents showed awareness that a behavioural switch occurred sometime after immersion in water from an initial active coping style (swimming, climbing and struggling) to increasing bouts of passive coping until finally a motionless state of floating was achieved.

In this article, we report data on a representative sample of recent FST publications in order to learn more about the current trends in the interpretation of FST behaviour. A second set of data that we collected was randomly extracted from journals with a high frequency of FST articles. These data served to investigate the overlap in outcome among several presumed behavioural read‐outs for depression and anxiety, such as, for example, the FST, tail suspension test (TST), sucrose preference test (SPT) and elevated plus maze. Based on these data, we asked the Editors of the selected journals for comment and we are grateful for their rapid and constructive cooperation.

In the final section of this article we first summarize the FST science of the 1980s when the test was scrutinized with pharmacological and procedural methods to figure out what was actually measured. This research led at the time to the conclusion that the FST had validity to identify analogues of tricyclic antidepressants, albeit with numerous false positives and negatives, and that endocrine (glucocorticoids, opioids) and metabolic factors have a key function in the consolidation of acquired immobility. Then, we highlight research of the past decade on the mechanistic underpinning of the immobility response the rodent displays gradually during coping with the inescapable forced swim stressor. The recent findings highlight the *top‐down* control of prefrontal‐limbic circuitry and *bottom‐up* action of glucocorticoids in the selection, consolidation and retention of a passive coping style with the inescapable stressor (Johnson et al., 2016, 2018; Lingg et al., 2020; Radley & Johnson, 2018; Wood et al., 2018). Our contribution to this special issue on Stress, Brain and Behavior is a tribute to the late Bruce McEwen, pioneer in Psychoneuroendocrinology.

## 2 DATASET‐I: TRENDS IN USE AND INTERPRETATION OF THE IMMOBILITY RESPONSE

We collected a representative selection (Kotrlik et al., 2001) of the FST literature that was published between June 30, 2018 and June 30, 2020 complementing our earlier work Molendijk & de Kloet, 2015, 2019).

In 2015, we showed that from 1979 to 2014 the use of the FST became increasingly popular and that in more and more articles the immobility response was labelled as *depression‐like behaviour* or in related terms such as *helplessnes*s or *despair* (Molendijk & de Kloet, 2015). In analyses on the use of the FST over the years 2014–2018 we confirmed its popularity of use. Although the majority of articles still interpreted the immobility response as *depression‐like behaviour*, the trend of increased frequency of labelling immobility as such was not present anymore. Labelling immobility as a *coping style, strategy* or even as a *learned response* became more popular over the years 2014–2018 (Molendijk & de Kloet, 2019).

**FIGURE 1:**
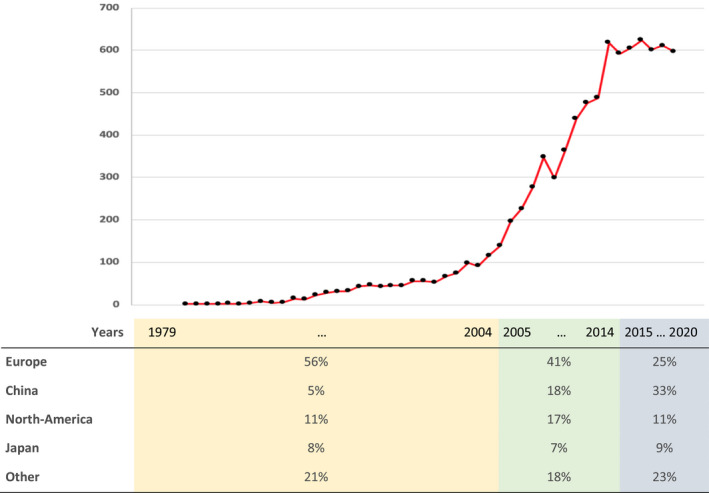
Number of publications in PUBMED reporting results from the forced swim test by year and the relative contributions to this number by continent or country (+ rest category) per time interval

We estimate that between June 30, 2018 and June 30, 2020, 1,210 articles have been published that used the FST (601 in 2018, 611 in 2019, and based on the current numbers, 597 in 2020). These numbers indicate the break of a trend. The number of yearly published papers that used the FST has increased since the inception of this test in 1978 but from 2014/2015 on, the curve started to flatten (see Figure 1). A statistical assessment confirms the break of a trend as correlations between year and the yearly number of published articles that used the FST differ significantly over the time frame 1979–2014 (*r* = 0.85, *p* < 0.01) relative to the time frame 2014–2020 (*r* = −0.19, *p* = 0.68; *z*_diff_ = 2.72, *p* < 0.01).

We randomly selected 221 articles from the 1,210 articles that were published between June 30, 2018 and June 30, 2020. Nine of the selected articles were not eligible for the current purposes as they did not report original data. The remaining 212 articles were analysed on how the main outcome of the FST experiment, immobility, was interpreted. There were no significant trends in the labelling of immobility over the time frame June 30, 2018 to June 30, 2020 (Lambda = 0.09, *SE* = 0.06, *p* = 0.12). So, the newly collected data was analysed as a single unit.

Most studies were performed in China (34%), followed by other Asian countries, Europe and the United States of America. The relative contribution of Chinese laboratories to the FST literature increased six‐fold over the years. The contribution of laboratories from South‐America and Asia—other than China—also increased. The relative contribution of European laboratories decreased to <50% (see Figure 1). The relative contributions of laboratories from North‐America and Japan, that also have a large FST output, largely remained the same over the years.

In 59% of the articles, the mouse served as study subject and in 40% the rat. Two experiments were performed with hamsters. In 80% of the cases, male animals were used. A single swim session was applied in 61% of the articles and 90% of the swim sessions took place in water that was between 24 ± 2°C, as is specified in the original protocol. About half of the manipulations were pharmacological in nature (51%), followed by exposure to food items or herbs and genetic manipulations. In 40% of the cases, the animals were stressed (e.g., restraint stress, social defeat stress) prior to be subjected to the FST.

In 58% (SD = 6%) of the articles, immobility was scored as *depression‐like behaviour* and in 30% (SD = 1%) as a *response on a compound that potentially has antidepressant properties* (note that this category includes both pharmacological‐ and non‐pharmacological agents). Five % (SD = 1%) of the articles scored immobility as coping and 9% (SD = 1%) of the articles, scored immobility in a different way (e.g., as *immobility* or as *anxiety*). This pattern differs from that observed in the time‐frame 2014–2018 (*χ* ^2^ = 8.10, *df* = 3, *p* = 0.04). Currently, 15% fewer articles score immobility as *depression‐like behaviour*, while interpretations as *coping* and *other* are more often used (from 2% to 4% and 8% to 10% respectively), a trend that confirms our previous analysis over 2014–2018 (Molendijk & de Kloet, 2019). The interpretation of immobility as a response to antidepressants also is used more frequently in recent years (30% of the cases), relative to 2014–2018 (19% of the cases). This latter observation seems due to a trend in investigating food types or herbs for their potential *antidepressant* properties. This is done in about 10% of all the FST articles that were published in the past 2 years, whereas in previous years this type of research hardly was reported at all.

The interpretation of immobility was related to geographic region where the experiment was performed (Goodman and Kruskal tau (*τ*) = 0.05, *SE* = 0.02, *p* < 0.05). In experiments that were performed in China, immobility was relatively often interpreted as *depression‐like behaviour* while in experiments that were performed in North‐America or Europe, the immobility response was relatively often interpreted as a *response to an antidepressant*. This is relevant for understanding trends in the interpretation of immobility over time since the relative contribution of countries and continents to the FST literature also changed over the years (see Figure 1).

Researchers were more likely to label the immobility response as *depression‐like behaviour* in case the FST followed exposure to stress (*τ* = 0.02, *SE* = 0.01, *p* < 0.05). Over the years, more experiments were published that exposed animals to stress (e.g., restraint stress). In the period 2015–2018 this was done in 24% of studies while in the period 2018–2020 it was done in 40% of studies. Rats were more likely to be involved in experiments in which *depression‐like behaviour* was modelled whereas mice were more often used in experiments in which the antidepressant capacity of compounds was examined (*τ* = 0.05, *SE* = 0.01, *p* < 0.01). The use of type of animal did not change over time.

The use of single session versus a two‐sessions FST and sex of the animal were not associated with the interpretation of immobility, neither were water temperature, statistical significance of the experiment and impact factor of the journal in which the findings were published.

## 3 DATASET‐II: WITHIN‐STUDY OVERLAP IN OUTCOME OF SEVERAL BEHAVIOURAL READ OUTS

We gathered a second dataset to investigate the within‐study overlap in outcome of several behavioural read‐outs that are often combined with the FST for assessment of depression‐like phenotypes. We also incorporated the order in which the various tests were performed in the assessment. The data set was a random sample of FST articles that were published between June 30, 2010 and June 30, 2020 in dedicated journals that we selected because of their focus on behavioural phenotyping rather than pharmacological screening. These selected Journals were *Behavioral Brain Research*, *Biological Psychiatry*, *Neuroscience*, *Physiology & Behavior* and *Psychoneuroendocrinology*. The second data‐set also served as a starting point for a discussion with the Editors of these journals on the interpretation of the FST data.

We estimate that between June 30, 2010 and June 30, 2020, these five journals together published 1,302 articles in which the FST was used. Table S1, provides the number of published articles per journal over the past 10 years and the frequency of use of other behavioural read‐outs alongside the FST. From each Journal we randomly selected a sample of about 22% of the published papers for further analyses. Overall, in 41% of the studies animals were subjected to either the TST or SPT or both these tests (range 30%–66%) alongside the FST. Tests for anxiety‐like behaviour were performed in conjunction with the FST in 55% (range 48%–61%) of the articles. On average, 74% of the FST experiments assessed whether plain differences in locomotor activity resulted from the experimental manipulations (range over journals is 72%–83%) and hence could explain the results derived in the FST (see Table S1). Table S2 provides summary statistics on the labelling of the immobility response, overall and per journal.

One of the questions that we wanted to answer was on the within‐study overlap between FST outcome and the outcomes derived from the TST, the SPT and some behavioural tests for anxiety‐like behaviour; the Elevated Plus Maze (EPM), the Light‐Dark Box (LDB), and the Open Field Test (OFT). Box S1 provides basic information on these tests.

In the FST, animals cope with an inescapable stressor, initially in an active way by swimming, struggling and climbing which is then interrupted by increasingly more periods of immobile floating that also increase in length of time (de Kloet & Molendijk, 2016). Given conceptual similarities between the TST and the FST (see Box S1), we expected that behavioural responses to these tests would overlap. To statistically address this, we cross‐tabulated the variables *relative active*‐ or *passive coping* in the FST and the TST. We speak about *relative* because the changes in coping styles as they are observed in the experiments are relative (e.g., stressed vs. non‐stressed).

The SPT is conceptually different from the FST (see Box S1). We have no specific ground to belief that this read‐out is associated with displaying an active‐ or a passive coping style in the FST. Statistically, we investigated the overlap in behavioural responses on these tests by cross‐tabulating the variables *relative active*‐ or *passive coping* in the FST with a variable coding for whether *sucrose preference relatively increased or not* in the SPT. Again, we speak of *relative* as the responses on the tests usually follow from a manipulation (e.g., treated with drug X vs. placebo).

The EPM, LDB and OFT are used as a read‐out for anxiety‐like behaviour (see Box S1). Assessment of anxiety is based on similar principles in these tests; the tendency to seek safety versus the tendency to explore (Himanshu & Sarkar, 2020). We do not have specific expectations with regards to the overlap in the behaviour that is displayed by the animal in either one or in a combination of these tests and the behaviour that is displayed in the FST. Overlap in these two types of tests was statistically assessed by cross‐tabulating the variables on whether the animals expressed a *relative active*‐ or *passive coping* style in the FST versus anxiety like‐behaviour in the tests that were performed to assess this.

Table 1 displays the percentage of overlap in behavioural responses to the tests. Manipulations yielded corresponding passive or active coping response in the FST and the TST (Mantel‐Haenszel odds ratio [OR] for corresponding results over tests = 10.72 [95% confidence interval (CI) = 2.04 to 56.60, *p* = 0.005]). The coping style that was displayed in the FST was not associated with sucrose preference (OR = 0.75 [95% CI = 0.26–2.15, *p* = 0.59]) and also not with anxiety‐like behaviour (OR = 0.89 [95% CI = 0.37–2.10, *p* = 0.78]).

**TABLE 1:** Within‐study overlap between FST outcome and the outcomes derived from the other tests

	FST/TST^1^	FST/SPT^2^	FST/ANX^3^
Overall	*n* = 44 I (68%), II (14%), III (18%)	*n* = 73 I (36%), II (23%), III (41%)	*n* = 139 I (27%), II (43%), III (30%)
Behavioral Brain Research	*n* = 15 I (54%), II (13%), III (33%)	*n* = 17 I (24%), II (24%), III (52%)	*n* = 47 I (30%), II (40%), III (30%)
Biological Psychiatry	*n* = 15 I (80%), II (13%), III (7%)	*n* = 19 I (32%), II (26%), III (42%)	*n* = 30 I (30%), II (40%), III (30%)
Neuroscience	*n* = 6 I (83%), II (0%), III (17%)	*n* = 14 I (36%), II (21%), III (43%)	*n* = 32 I (28%), II (47%), III (25%)
Physiology & Behaviour	*n* = 4 I (75%), II (25%), III (0%)	*n* = 15 I (53%), II (20%), III (27%)	*n* = 29 I (35%), II (37%), III (28%)
Psychoneuroendocrinology	*n* = 4 I (50%), II (25%), III (25%)	*n* = 8 I (38%), II (24%), III (38%)	*n* = 19 I (11%), II (47%), III (42%)

Note

Outcome categories: ^1^I = passive or active in both tests; II = not clear or a mix between I and III; III = passive in one test and active in the other test or active or passive in one test and no change in the other test. ^2^I = passive in the FST and decreased sucrose preference or active in the FST and increased sucrose preference; II = not clear or a mix between I and III; III = passive in the FST and increased sucrose preference or in the FST and decreased sucrose preference or active or passive in the FST and no change in sucrose preference. ^3^I = passive in the FST and increased anxiety or active in the FST and decreased anxiety; II = not clear or a mix between I and III; III = coping in the FST and decreased anxiety or active in the FST and increased anxiety or active or passive in the FST and no change in anxiety.

Abbreviations: FST, forced swim test; SPT, Sucrose Preference Test; TST, tail suspension test.

In by far most studies in which rodents were subjected to the TST and/or SP alongside the FST, the latter was applied as the closing test of the sequence (75%, range over journals 58%–83%, see Table S1). This was also true in case the FST was applied together with the EPM, LDB and/or OFT, also here the FST was most often the final test that was run (87%, range over journals 80%–92%, see Table S1). Note that these estimates are based on a sub‐sample of studies as in 51% of the FST/TST/SP studies and 40% of the FST/EPM/LDB/OFT studies, authors did not report the sequence of test administration. Authors did often refrain from a justification or explanation for applying this sequence of test administration. In some cases (about 5% of the studies), it was mentioned that different cohorts of rodents are exposed to the FST and the other tests, given the invasive nature of the FST.

While going through the articles, we noted some apparent changes in the FST literature over the years, which we want to share with the readership. These changes include an increased use of the so called 2‐stage FST. In this variant first chronic stress exposure was used to increase FST immobility as presumed measure for depression‐like behaviour. Next a (pharmacological) manipulation was applied as a test for its antidepressant potential. Another trend is the assessment of the effects of dietary components, herbs or microbiota manipulation on immobility in the FST. A third trend is the increased use of the FST as a read‐out for the effects of ketamine or norketamine. Box S2 describes these trends in more detail and presents an exemplary paper on each trend.

## 4 EDITORS OPINION

The second data‐set also formed the starting point for an exchange of thoughts with the Editors‐in‐chief of the journals that report most often FST research. We sent them statistics on the use and interpretation of the FST in their journal (see Tables S1 and S2) along with the question on their *point of view as Editor‐in‐Chief regarding submissions in which FST performance is considered a depression‐like behaviour*. Our survey had a 100% response rate. The responses by the Editors—often formulated after elaboration with Section‐Editors—are summarized in Box 1, alongside their opinion on the use of the FST as a read‐out for depression‐like behaviour.

**What is your point of view as Editor‐in‐Chief regarding submissions in which FST performance is considered a depression‐like behaviour?**

***Behavioral Brain Research*** *—Editor‐in‐Chief:* Stephen Maren *Phd:* The Behavioral Brain Research Editors do not take a position on the interpretation of performance in specific behavioral tasks, including forced swim.

Opinion on the use of the FST as a read out for depression‐like behavior: No opinion provided.

***Biological Psychiatry*** *—Editor‐in‐Chief:* John Krystal *MD Phd, Section Editor* Eric Nestler *MD Phd:* We try not to micromanage our reviews. I don't want to be in the position of censoring what our scientists want to say in their papers.

*Opinion on the use of the FST as a read out for depression‐like behavior:* [We] still have so many studies where floating is described as depression‐ or despair‐related. That's not good for the field.

***Neuroscience*** *—Editor‐in‐Chief:* Juan Lerma *Phd and section Editors* Francesca Cirulli *Phd* and Julie Fudge *Phd:* Our position is that unless the scientific community (authors and reviewers) don't mature this knowledge we are not so sure that the journal should take on the mission of, for example, rejecting papers on the basis of how the FST is used.

*Opinion on the use of the FST as a read out for depression‐like behaviour:* The FST per se is not so informative about depression.

***Physiology & Behavior*** *—Editor‐in‐Chief:* Thomas Lutz *Phd* and *field Editors*: Until now we leave to the referees’ criteria to evaluate how adequate it is to use FST in the context of depression, and whether the conclusions attained are sound or not, that is, we support the independence of the reviewers to judge to the best of their knowledge the adequacy of methods used in a paper.

*Opinion on the use of the FST as a read out for depression‐like behavior:* Overall, … the test is often over‐interpreted. … and … its translational value may be much lower than originally thought.

***Psychoneuroendocrinology*** *—Editors‐in‐Chief:* Isabella Heuser *Phd* and Robert Dantzer *Phd:* Our policy is to draw the attention of the authors to the NIMH considerations on the use of animal behavior for studying psychiatric disorders (https://grants.nih.gov/grants/guide/notice‐files/NOT‐MH‐19‐053.html) and to remind them of alternative explanations to immobility in the FST. We refrain from being prescriptive.

*Opinion on the use of the FST as a read out for depression‐like behaviour:* The FST is what we call a behavioral test tube which we all know is sensitive to antidepressants, therefore its high predictive value when used for screening drugs, but of which the face validity as depression model is close to zero.

From the responses we can conclude that the Editors in general do not interfere in or “micromanage” FST submissions as long as the science is judged sound by the reviewers. Editor's opinions on the FST as a read‐out for depression‐like behaviour are outspoken and resonate that the FST is very limited in its capacity to model depression‐like behaviour. The Editor of *Psychoneuroendocrinology* who informed authors about the NIMH notice on animal behaviour noted in 2011 (Dantzer et al., 2011) page 427 “The number of original research articles describing antidepressant or depressant behaviour phenotypes induced by specific gene or environmental alterations is still increasing even in high impact factor journals. This trend is evident in the absence of any obvious link between the condition under study and depression.” See also a quote from an authoritative paper on a translational framework for animal research of psychopathological states in depression (Pryce & Seifritz, 2011) page 318 “As will be clear from the central theme of this paper, the proposal that despair resulting from an uncontrollable stressor mediates mouse behaviour in the FST and TST is problematic.” The authors continue by articulating these problems while referring to immobility in the FST as a phase of motor inactivity.

The classic on animal models of neuropsychiatric disorders (Nestler & Hyman, 2010) is a 'must read' for all students in translational neuroscience. In their analysis the authors describe in a concise way not only the almost impossible challenge for using DSM criteria for generation of an animal model of mental disorders, to achieve construct validity, but also guidelines for criteria for description and focus to which the animal models should obey. The authors conclude among others on page 1,167: “We would now eschew the all too common practice of using black box behavioral tests developed as drug screens as if they confer face validity. A corollary of this is that tendentious anthropomorphizations, such as describing responses in the FST as behavioral despair, should be avoided in the scientific literature.”

In our analysis of the publication pattern of the FST application in dataset I, we noted that the use of the FST for antidepressant drug screening was further diminished, but that its application for phenotyping of animal behaviour remained popular and was extended the past years to test interventions in stressed‐out animals. These interventions included treatment with dietary and microbiotic components, herbs and new rapidly acting antidepressants such as ketamine. Effects of environmental or behavioural enrichments were also included. The idea was that chronic stress exposure would have deleterious influences as judged from, for example, increased immobility during forced swim exposure and that any treatment resulting in reduced immobility would qualify as an inroad towards boosting of resilience. However, it is also has become obvious that such interpretations based on a single behavioural test were to reductionistic. Increasingly, integral behavioural *z*‐scores are being used by combining among others the outcome of the FST, SPT, social interaction and fear conditioning paradigms (Guilloux et al., 2011). Except for the TST and FST our analysis of the literature provided little congruence in the outcome of these tests. For more information on behavioural *z*‐scores, we refer to (Guilloux et al., 2011) and (Labots et al., 2018) and to Box S3.

## 5 The FST measures coping and adaptation

Porsolt's claim of the FST as behavioural despair model (Porsolt et al., ,^1977^, ^1978^) was based on an anthropomorphic interpretation of immobility in terms of …*giving up hope to escape*. In the 1980s more and more facts were provided that made immobility as measure for despair unlikely. It was established for instance that animals familiar to the test were more immobile (Borsini et al., 1986). Furthermore, if animals were exposed to water with a temperature of 19°C at the initial test, they became more immobile if re‐exposed to 25°C (Reul et al., 2015). Also, offering an escape route during retest did not reduce immobility scores (O’Neill & Valentino, 1982). Moreover the immobility response could not be generalized to other immobility (freezing) responses during inescapable electric shock conditions which argues against a “learned helplessness” interpretation (Maier & Seligman, 2016). Finally, although a seemingly awkward criterion that perhaps would not meet today's animal welfare regulations, Nishimura et al. (1988) established that rats engaged in immobility did not sink as readily as the swimming rats suggesting that because of this the rodents that “*float longer probably live longer…*”. See also the Essentials of Sea Survival (Golden & Tipton, 2002).

Tricyclic antidepressants interfered with retention of the acquired immobility response (De Pablo et al., 1989). Alternatively, SSRI’s did not interfere with immobility and thus were considered *false negatives* (Porsolt, 1989). A long list of compounds including amphetamine (Porsolt et al., 1977), GABA agonists (Mombereau et al., 2004), and anticholinergic agents (Weiner et al., 2003) all qualified as *false positives*. However, exercise which increases glucocorticoid secretion and is thought to enhance resilience and thus acts as some sort of anti‐depressant also increased immobility thus qualifying as a *false negative* (Collins et al., 2009). Rapidly acting ketamine (Khakpai et al., 2019), electroshock exposure (Li et al., 2007) and deep brain stimulation (Hamani et al., 2010) decrease immobility time and thus were interpreted as supportive for the “antidepressant” line of reasoning.

In conclusion, the Porsolt swim test, thus had some validity in the search for new compounds with an antidepressant potential, but the numerous false negatives and positives limited its application. The consensus at the time was that the FST unlikely modelled behavioural despair, depression‐like behaviour or helplessness, which were felt to be anthropomorphic interpretations of animal behavior. Moreover as was pointed out by Porsolt himself: the behaviour measured was a dependent variable of the experimental condition (Castagné et al., 2009). Accordingly, the view by Hawkins became leading in the 1980’s that the progressive periods of immobility towards the end of the 15‐min initial test including the rescue of the animal is an adaptive response “without the energy expenditure required in swimming” (Hawkins et al., 1978).

However, this knowledge generated in the 1980s was largely brushed away with the FST revival that occurred around 2000 (see Figure 1) as a rapid bio‐assay for “despair” and depression‐like behaviour when pressure was building up to phenotype a plethora of models generated by exposure to a wide variety of stressors and/or genetic deletions of all kind (Cryan & Mombereau, 2004; Cryan et al., 2002; Dalvi & Lucki, 1999; Porsolt, 2000; Slattery & Cryan, 2012).

## 6 GLUCOCORTICOIDS PROMOTE MEMORY CONSOLIDATION OF PASSIVE COPING

Our interest in the FST was raised in the mid 1980s because of the possibility this test offered to assess the role of glucocorticoids in coping with an inescapable stressor. In 1983, Don Jefferys and John Funder discovered that adrenalectomized (ADX) rats could acquire immobility over the 15 min initial test at a rate that was indistinguishable from adrenally‐intact animals, that is, from 30% immobility the first 5 min of FST exposure, to 50% from 5 to 10 min and about 70% at 10–15 min. ADX animals were however unable to retain acquired immobility in the 5 min retest 24 hr later. While intact animals showed again 70% of the time immobility, that of ADX animals was reduced to only 28% at retest (Jefferys et al., 1983).

Veldhuis et al. (1985) reproduced Jefferys data and also showed that microgram amounts of dexamethasone (or of the pure glucocorticoid receptor [GR] agonist RU28362) or milligram (mg) amounts of corticosterone, but not progesterone, aldosterone or deoxycorticosterone, administered 15 min *after* the initial test entirely reinstated floating to the levels observed in adrenally‐intact animals; the steroids were not active if administered 1 hr *before* the retest (Jefferys et al. 1983; Veldhuis et al. 1985). The anti‐glucocorticoid RU486 (mifepristone) given in mg amounts at 5 min *before* initial testing interfered with retention of acquired immobility and scores at retest resembled those of ADX animals (Jefferys & Funder, 1987; Veldhuis et al. 1985). This effect exerted by systemic RU486 was mimicked with an infusion in the hippocampal dentate gyrus of a 100,000‐fold lower dose in the low nanogram range. In the paraventricular nucleus (PVN) the GR antagonist was behaviourally inactive, but evoked a profound hypothalamic–pituitary–adrenal (HPA)‐axis response (Dalm et al., 2019; de Kloet et al., 1988). These data clearly point to a GR‐mediated action on consolidation and retention of the acquired immobility response.

**FIGURE 2:**
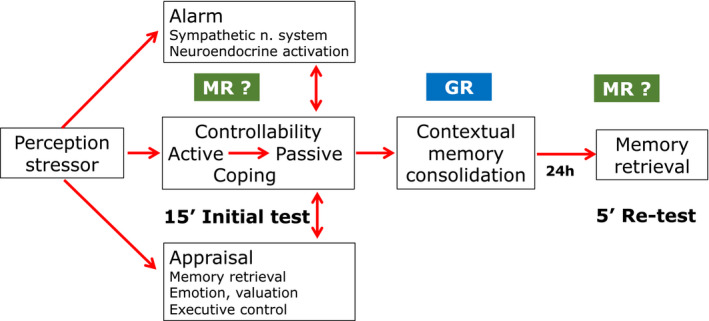
The inescapable forced swim stressor. Upon perception of the *inescapable* forced swim stressor an immediate alarm reaction is triggered that activates the sympathetic nervous system and a neuroendocrine cascade, the hypothalamic–pituitary–adrenal (HPA) axis, which interact with appraisal process underlying controllability of the situation. In the initial test the rodent displays bouts of increasing frequency and duration of motor inactivity until the animals floats immobile. . GR activation promotes consolidation and retention of this passive coping response, which is displayed again at re‐test (de Kloet et al., 1988; Gutierrez‐Mecinas et al., 2011; Reul et al., 2015). A role of MR in selection of coping style with the inescapable stressor, and during retrieval during the retest has not been established yet. During processing of *escapable* and *controllable* stressors (non)genomic MR functioning regulates risk assessment, selection of coping style and memory retrieval (Oitzl & de Kloet, 1992; Schwabe et al., 2010, 2013). MR blockade attenuates violent aggression (Kruk et al., 2013) and is anxiolytic (Korte et al., 1995). Genomic GR activation promotes contextual memory consolidation Upon a new encounter MR activation promotes again memory retrieval of the experience stored in the memory (Oitzl & de Kloet, 1992; Roozendaal et al., 2009)

The pure MR antagonist RU28318 was ineffective on consolidation and retention of passive coping with the inescapable FST stressor if given briefly before or after the initial test (de Kloet et al., 1988). Only two studies showed that pretreatment with spironolactone could interfere with corticosterone effects on immobility in a serotonin‐dependent fashion (Mostalac‐Preciado et al., 2011; Wu et al., 2013). Daily RU486 for 5 days also interfered with immobility if the last injection was given 1 hr prior to the initial test (Dalm et al., 2019; Solomon et al., 2014; Wulsin et al., 2010). This effect may be either due to chronic blockade of the GR known to result in a change in setpoint of the HPA‐axis or involve brain MR which remain available for circulating corticosterone in the presence of the antagonist (Dalm et al., 2019). In “escapable” behavioural paradigms characterized by a sense of controllability MR blockade was found to interfere with appraisal processes, risk assessment, selection of coping style, or with retrieval (de Kloet et al., 2018; Harris et al., 2013; Schwabe et al., 2010; Souza et al., 2014). Systematic studies on the role of MR in the transition of active to passive coping with the “inescapable” stressor in the initial test or on the retrieval of acquired immobility in the retest are unfortunately lacking (Figure 2).

Subsequent studies showed that also adrenomedullectomy interfered with the retention of immobility, be it transient: recovery of retention was observed after some weeks while the effect of ADX persists (Veldhuis et al. 1985). In line with a role of the adrenal medulla, Jefferys demonstrated that the retention deficit of ADX animals also could be restored with an enkephalin analogue or with dynorphin (1–17) suggesting implication of a κ‐selective opioid pathway (Jefferys & Funder, 1987; Jefferys et al., 1984). In subsequent studies, it was found that thyroid hormone also could reverse the behavioural deficit of ADX animals (Jefferys & Funder, 1993). Twenty‐four hours of food deprivation interfered with retention of immobility and this deficit could be restored with dexamethasone. Moreover the behavioural deficits after ADX as well as food deprivation could both be corrected (i.e., by restoring immobility at retest) with glucose administration (Jefferys & Funder, 1991). Accordingly, it was concluded that for coping with the inescapable forced swim stressor a “complex interplay of endocrine and metabolic factors” is necessary to achieve retention of the acquired immobility response.

With the advent of genetic modification of the mouse brain GR, some of the generated mutants were tested for their immobility response in the FST (Laryea et al., 2015). The results were largely in line with the pharmacological findings. For instance, the GR^Nes/Cre^ total brain knockout (Tronche et al., 1999) displayed *reduced* immobility at retest mimicking the deficit in retention of acquired immobility of the ADX animals. Forebrain knockout animals (GR‐FBKO) showed *increased* immobility (Boyle et al., 2005) which can be explained by the sparing of hypothalamic, central amygdala and brain stem neurons expressing GR in this model. Pituitary‐selective GR POMC^Cre^ deletion showed a similar phenotype as the whole brain GR knock out, which suggests that also early life effects need to be taken into account (Schmidt et al., 2009). GR deletion from glutamatergic‐ rather than GABA‐ergic neurons caused hyperactivity of the HPA axis and reduced anxiety‐related behaviours (Hartmann et al., 2017). Although not investigated one would predict in these lines an FST‐ADX or GR^Nes/Cre^ phenotype. A recent study based on a CRISP/Cas9 approach targeting the rat prelimbic mPFC neurons (pl PFC CaMKIIa GRKO) did not affect immobility, but revealed minor sexual dimorphic effects with the males showing increased aspects of an active coping style with the inescapable stressor (Scheimann et al., 2018).

The pioneering research by Hans Reul and colleagues has revealed aspects of the mechanism underlying the glucocorticoid action in memory consolidation. They discovered that in discrete hippocampal dentate gyrus neurons the corticosterone rise during the initial 15 min of forced swim exposure causes activation of GR in synergism with the NMDA‐induced pERK1/2—MAPK pathway. Within 15 min this apparent non‐genomic GR and nuclear kinases MSK1/2and Elk1 synergism cause Histone‐ phosphorylation and acetylation resulting in epigenetic changes, induction of the immediate early genes c‐Fos and Egr‐1 and rapid chromatin reorganization. Indeed, upon genetic deletion of one of the nuclear kinases the GR‐dependent retention of acquired immobility does not occur. As shown by c‐Fos expression this GR‐driven molecular cascade in crosstalk with ERK1/2–MSK1–Elk‐1 signalling occurs in only a few dispersed dentate cells (Gutierrez‐Mecinas et al., 2011; Reul et al., 2015). Interestingly, the ERK1/2—MAPK pathway appeared also a GR‐mediated target of a tissue plasminogen (tPA)‐BDNF signalling pathway relevant for contextual fear memory consolidation (Revest et al., 2014).

Transcriptome analysis of laser‐dissected dentate gyrus neurons did not reveal much changes in gene expression after chronic exposure to stress or corticosterone (Datson et al., 2013). Profound changes relative to control animals were, however, observed if such chronically treated animals were exposed to an acute heterotypic stressor, to corticosterone or a GR antagonist. Thus, a CREB‐BP signalling pathway in the dentate gyrus responded to GR blockade in chronically stressed rats (Datson et al., 2012). Interestingly, acute exposure to corticosterone or a forced swim stressor showed activation of the NFκB signalling pathway, and of HDAC involved in epigenetic regulation immediate early gene production and chromatine reorganization (Datson et al., 2013; Gray et al., 2014; Polman et al., 2012). In support of these findings contextual memory consolidation appeared causally linked to CREB target genes identified with expression profiling in an engram localized in sparsely distributed dentate gyrus neurons (Rao‐Ruiz et al., 2019).

In conclusion, coping with the inescapable forced swim stressor rather depends on a ‘complex interplay of endocrine and metabolic factors’ which is in support of Hawkin's line of reasoning (Hawkins et al., 1978). The pharmacological and gene deletion studies demonstrated that GR activation in hippocampus is a necessary step in the consolidation and retention of the passive coping style, which is also a consistent finding in other behavioural paradigms (de Quervain et al., 2009; Kaouane et al., 2012; Oitzl & de Kloet, 1992; Oitzl et al., 2001). After examining the action of glucocorticoids on memory consolidation of the passive coping style, the molecular signature of GR activation appeared localized in an engram of sparsely distributed dentate gyrus neurons (Gutierrez‐Mecinas et al., 2011; Reul et al., 2015).

## 7 mPFC PROJECTOME AND PASSIVE COPING

Further support for effects of the forced swim on c‐Fos immediate early gene expression in the dentate gyrus came from Simona Cabib's group by comparing performance of mice of the DBA2 versus the C57Bl6 strains. The C57Bl6 mice show a profound HPA axis activation and glucocorticoid secretion upon exposure to forced swim. The mice readily assume an immobile floating position during the 15 min initial test which is retained in the 5 min retest 24 hr later. The passive C57Bl6 mice showed increased c‐Fos expression in the hippocampus, which upon lesioning results in an abolished retention of immobility (Colelli et al., 2014). In contrast, the DBA mice remained swimming and otherwise active when facing the inescapable stressor and showed that c‐Fos activation was most prominent in the dorsolateral striatal (DLS) dopaminergic neurons. Lesioning of the left DLS or treatment with D2 antagonists interferes both with memory performance of the DBAs. Retention of acquired immobility of the DBAs was also abolished—as was previously shown (Jefferys & Funder, 1993)—by food deprivation which is known to downregulate the DLS‐D2 receptors (Campus et al., 2017; Fiore et al., 2015).

Other pathways involved in passive and active coping with the forced swim stressor were identified by optogenetic manipulation of the ventral tegmental area (VTA) A10 meso‐cortical dopaminergic reward and motivation circuitry. Stimulation of the VTA neurons was capable to enhance active coping, while inhibition of these neurons increased the passive immobility response (Tye et al., 2013). These optogenetic findings match the activity of the VTA‐DA neurons during the FST. Initial exposure to the forced swim causes active coping and an increase in DA release. This turns into a decrease in VTA‐DA release once immobility is acquired (Fiore et al., 2015). VTA‐DA function also decreases after exposure to chronic unpredictable stressors in parallel with increased FST immobility, which could be reversed with optogenetic activation of the VTA‐DA neurons (Belujon & Grace, 2017; Tye et al., 2013). Finally, in a study designed to select animals based on FST performance, it was found that the passive copers had increased dopamine turnover in the VTA‐amygdala pathway (Wisłowska‐Stanek et al., 2018). This finding suggests that it is important to identify the projection of the optogenetic manipulated VTA neurons.

In the previous two paragraphs, the immobility response or passive coping style appeared to be linked to the hippocampal–amygdala and VTA dopaminergic circuitry which are substrates for valence, emotional and contextual aspects of an experience that is stored in the memory. Active coping relies on the dorsolateral (dl) striatum substrate that has a role in more habitual behaviour. But how are these regions implicated in the circuitry underlying active and passive coping responses that have been identified using other approaches? Notably, the mPFC projectome stemming from its ventral and dorsal neuronal ensembles has received much attention, since it underlies cognitive control of executive behaviours that are linked to periaqueductal gray (PAG) excitatory motor output. In fact, electrical or chemical stimulation of the ventrolateral (vl)‐PAG indeed evokes immobility. Moreover when faced with the inescapable physical or psychological stressor cFos activation of vl‐PAG neurons correlated with the evoked passive coping style (Bandler et al., 2000; Keay & Bandler, 2001). In parallel with these behavioural responses activation of specific neurons in the mPFC projectome controls, besides passive coping, also the activity of the HPA axis and the sympathetic nervous system (Johnson et al., 2016).

**FIGURE 3:**
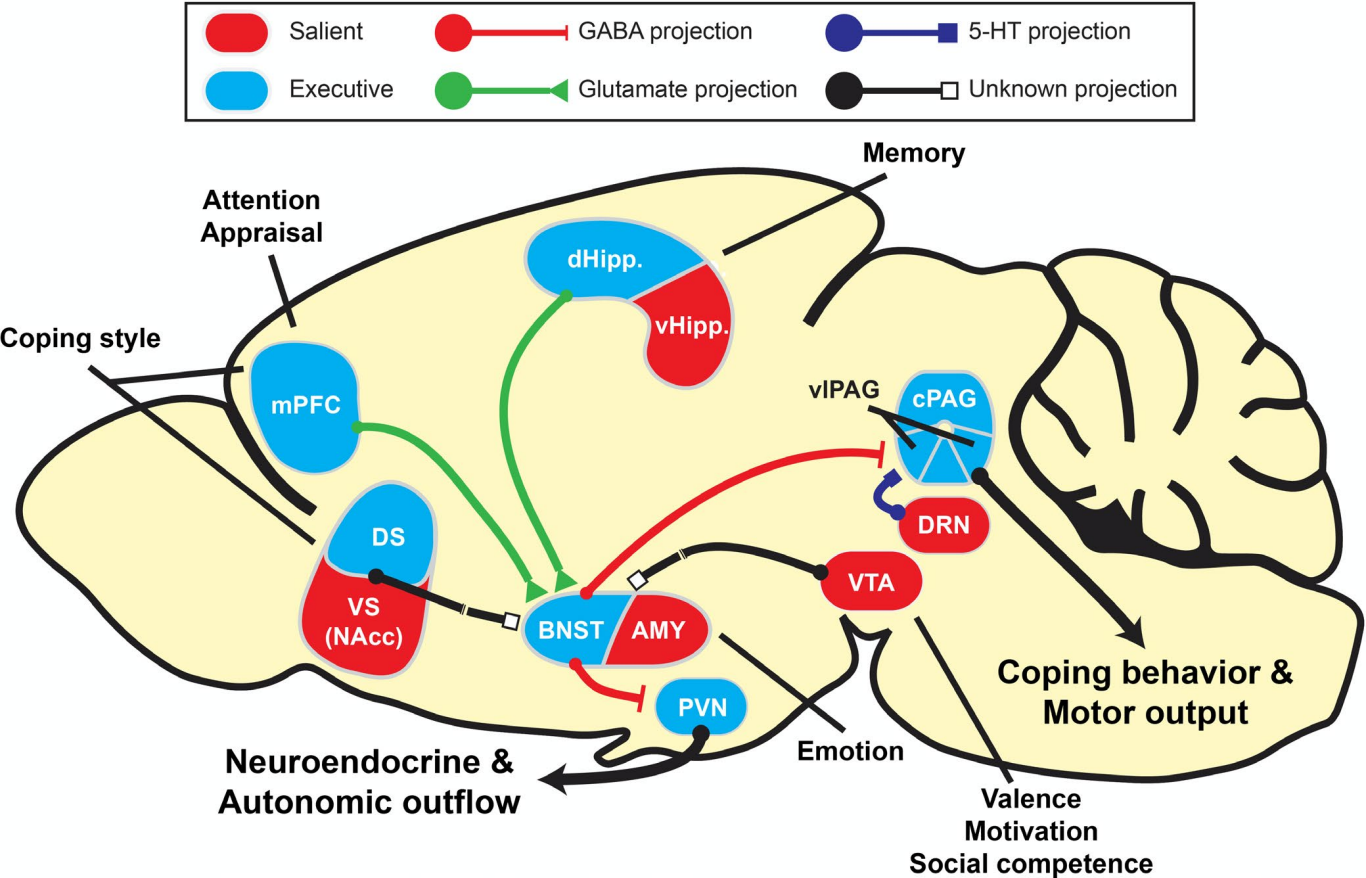
Stress coping circuitry. Stress‐coping circuitry as presented in a sagittal section of the rat brain with some selected regions involved in processing of salient information (red) and other regions in executive control (blue). During the experience of an inescapable stressor the excitatory output from the vl‐PAG is enhanced favoring a passive coping style. This effect may occur via either diminished GABA‐ergic inhibition of the avBNST hub as a result of reduced mPFC excitatory control and/or increased dorsal raphe serotonergic signals. Reduced mPFC control also disinhibits CRH neurons in the PVN resulting in hypothalamic–pituitary–adrenal axis activation and increased adrenocortical glucocorticoid secretion. Such reduced mPFC control was shown to occur under stress, but also can be manipulated optogenetically. The figure also shows modulatory influences from hippocampus, amygdala and the ventral striatum dopaminergic region involved respectively in contextual, emotional and valuation aspects of the stress experience. The circuitry seems to underly the progressive immobility displayed in the FST and TST as well as the immediate freezing responses in the shock‐prod defense burying, the “learned” helplessness model and various fear conditioning paradigms. One testable hypothesis is the passive coping style as the default survival mode under top‐down control of mPFC neuronal ensembles that gate vl‐PAG excitatory output underlying passive coping (Cabib & Puglisi‐Allegra, 2012; Johnson et al., 2018; Keay & Bandler, 2001; Lammel et al., 2014; Lingg et al., 2020; Radley & Johnson, 2018; Warden et al., 2012). Glucocorticoids act bottom‐up by promoting energy allocation by affecting mitochondrial function, by facilitating in coordination with aminergic inputs the selection of coping style and promoting rationalization, contextualization and memory storage in the executive brain circuitry as indicated in the figure (de Kloet et al., 2019; Hermans et al., 2014; Roozendaal & McGaugh, 2011; Scheimann et al., 2018, 2019; Weger et al., 2020; Wood et al., 2018). Figure adapted from (Douma & de Kloet, 2020). AMY, amygdala; BNST, Bed nucleus of the stria terminalis; dHipp., dorsal hippocampus; DS, dorsal striatum; GABA, γ‐aminobutyric acid; mPFC, medial prefrontal cortex; NAcc, nucleus accumbens; vl‐PAG, ventro‐lateral periaqueductal gray; PVN, paraventricular nucleus; vHipp., ventral hippocampus; VS, ventral striatum; VTA, ventral tegmental area

In their research, Jason Radley and colleagues have dissected in great detail how neuronal ensembles in a subregions of the dorsomedial PFC, i.e., the prelimbic (pl)‐PFC controls the activity of the neuroendocrine HPA axis and the vl‐PAG excitatory output as mechanism underlying passive coping (immobility) in the FST or TST as well as in defensive burying or the passive (inhibitory) avoidance test. Crucial in the control exerted by the pl‐mPFC output is the anteroventral (av) BNST hub from where GABA‐ergic projections innervate the hypothalamic PVN and the vl‐PAG (Radley & Johnson, 2018). Stimulation of the pl‐mPFC excitatory input to the av‐BNST activates its inhibitory GABA‐ergic output and results in suppression of HPA axis activation and a diminished immobility response. When pl‐mPFC input is attenuated as is the case by exposure to inescapable stressors, its excitatory input to the GABA‐ergic neurons is also attenuated which results in a less inhibitory signal to the vl‐PAG. Because of this reduced inhibition the excitatory outflow of the vl‐PAG is enhanced which then results in an increased immobility response as an experimental proof for the gating mechanism imposed by the mPFC‐avBNST input (Johnson et al., 2018; see Figure 3).

In their most recent experiments, Radley and colleagues (Lingg et al., 2020) succeeded to manipulate the BNST GABA‐ergic inputs to the PVN and vl‐PAG independently, while using the inhibitory (passive) avoidance response and adrenal corticosterone secretion as read‐outs. This fascinating study produced two main findings. First, retention of the passive avoidance response was enhanced when the GABA‐ergic input to the PVN was optogenetic attenuated. This decreased inhibition results in a corticosterone response that appeared indispensable for maintenance of passive avoidance possibly via an action on the amygdala and hippocampus. In contrast, stimulation of this input to the PVN had neither a neuroendocrine nor behavioural effect. Second, when the GABA‐ergic input to the vl‐PAG was separately stimulated, passive avoidance behaviour was impaired. In contrast, suppression of the GABA‐ergic signal—which was effective to the PVN‐ had no further effect on performance in the passive avoidance test. This study shows that the top‐down mPFC control of passive coping behaviour can cooperate with bottom‐up glucocorticoid action to facilitate memory storage of the immobility response (Lingg et al., 2020; see Figure 3).

The neurons of the vl‐PAG also receive input from brain stem aminergic neurons, notably the 5HT dorsal raphe neurons, the core system in Maier and Seligman's learned helplessness model. The raphe neurons, like the locus coeruleus noradrenergic neurons and n. tractus solitarii adrenergic neurons coordinate the function of the various PAG columns with numerous forebrain regions, allowing modulation in control of motor output during coping with stress (Maier & Seligman, 2016). Interestingly, through a series of ingenious experiments Maier and Seligman arrived at the conclusion that the immobility response during prolonged adverse conditions, that was originally labelled by them as learned helplessness, was not “learned” at all, but appeared the default mode. They found that already the anticipation to regain control of the stressor could activate mPFC inputs to the dorsal raphe nucleus‐vl‐PAG regulated motor output resulting in a reduction of passive coping styles (Maier & Seligman, 2016). Indeed optogenetic manipulation of neurons in the mPFC with long downstream efferents to the brain stem, notably, the dorsal raphe neurons, could affect passive coping with the forced swim stressor (Warden et al., 2012). See for a discussion on the role of mPFC neurons in control of stress‐coping the excellent review by Lammel and colleagues (Lammel et al., 2014).

Interestingly, in the previous paragraphs passive coping behaviour was shown to be affected by glucocorticoids and manipulation of the vl‐PAG excitatory output in several test situations. These include besides FST and TST also tests such as the defensive burying task, the fear conditioning paradigms recorded by “freezing” or as a passive (inhibitory) response measured as the delay to enter a compartment where previously a mild electric shock was experienced (Johnson et al., 2018; Lingg et al., 2020). Subsequently, once the behavioural response occurred the glucocorticoids appeared to facilitate consolidation and retention of the selected coping style in the memory. It is of interest to further explore the role of MR and GR in the mPFC‐BNST‐PAG pathway in escapable as well as inescapable stressors. Active coping with escapable psychological stressors is mediated by de dl PAG (Keay & Bandler, 2001). Interestingly, as early as 1981 it was found that extinction of the passive avoidance response, that relied on appraisal of a previously fearful context, was under control of the hippocampal corticosterone preferring MR (Bohus & de Kloet, 1981). This finding was later reinforced in transgenic mice that had their MR/GR balance genetically manipulated (Harris et al., 2013). MR was found to control risk assessment in an olfactory fear conditioning task (Souza et al., 2014) and in tasks designed to determine the switch between contextual and habitual memory performance (Schwabe et al., 2010). Other evidence points to a role of amygdala‐hippocampal GR in extinction of the immobile freezing response (de Quervain et al., 2019).

In conclusion, the abovementioned studies reveal a glimpse of future developments in the role of the mPFC projectome in top‐down control of coping with escapable and inescapable stressors. As was pointed out by (Giachero et al., 2019; Keay & Bandler, 2001) the PAG in‐ and outputs mediate a variety of coping reactions to different types of psychological and physical stressors. With the ascent of novel genetic and imaging technology's we will witness the coming years how neuronal ensembles in the various mPFC regions will exert their topdown cognitive control (Lammel et al., 2014; Terra et al., 2020).

## 8 GENETIC SELECTION OF COPING STYLES

The finding that the C57Bl6 and DBA2 mice show large differences in coping style linked to a different neuro‐anatomical substrate also raises the question whether passive and active coping styles have a a distinct genetic signature (Colelli et al., 2014). To that effect Henry and Stephens reported that when rats or mice are exposed to conspecifics the dominant animal fights, while the subordinate opponent displays “passive conservation withdrawal” if escape is not possible (Henry & Stephens, 1977). This passive response is viewed as a time of reduced responsiveness to environmental stimulation and quiescence allowing time for repairing damage such as wound healing in the aftermath of a fight. If such passive animals are selected and tested further, they do actually very well upon dispersal. This shows that the passive response is not an *innate* property, but depends on context, a hippocampus function, which led Koolhaas et al. to label this passive behaviour actually as the behavioural repertoire of a “reactive” (flexible) phenotype (de Boer et al., 2017; Koolhaas et al., 2010). In response to stress, glucocorticoid secretion in the subordinate phenotype is high while autonomic and immune activity is low in contrast to their dominant counterparts. The latter dominant mice were labelled with a “pro‐active” phenotype given their tendency to take pre‐emptive action as a means to gain control. The dominant phenotype is characterized by a high sympathetic and low glucocorticoid secretion, while displaying pro‐inflammatory and pro‐immune responses. The pro‐active animals tend to readily fall back on habitual “rigid” behaviours to deal with environmental challenges and flourish while in home territory. In other selection studies based on peripubertal stress the dominant animals displayed high GR expression in the amygdala (Papilloud et al., 2019).

The two phenotypes labelled in the Koolhaas studies as the “reactive” long attack latency (LAL) and “pro‐active” short attack latency (SAL) mice with regard to the time an attack is launched at the opponent, can be distinguished in other genetically selected lines also. Thus, the Roman high avoidance (RHA) versus low avoidance (RLA) rats (Steimer & Driscoll, 2003), the spontaneous hypertensive versus Wistar Kyoto rats (Armario et al., 1995) or the DBA versus the C57Bl6 mouse strains (Colelli et al., 2014) have in common that during genetic selection numerous properties are selected along with that particular criterion the experimenter is interested in. Nevertheless, The LAL, RLA, Wistar Kyoto and C57Bl6’s all express a flexible subordinate phenotype characterized by high stress‐induced corticosterone secretion which show upon exposure to the forced swim increased passive coping and thus superior retention of the acquired immobility response. In contrast the dominant pro‐active phenotype shows predominant active coping in the forced swim (Veenema et al., 2003). Of note, if C57Bl6 or BALBcJ mice are exposed daily for 5 days to the forced swim stressor, all mice display an increasing time of immobility, but no sign of a psychomotor, homeostatic or emotional dysfunction (Mul et al., 2016).

In conclusion, the mPFC projectome, involved in coordinating passive coping with physiological response patterns via the avBNST hub, may be fundamental for the extreme phenotypical differences in coping with stress that co‐exist in a normal population. The pioneering studies by Carmen Sandi's laboratory show that this is indeed the case in a process that is regulated by GR in the VTA and amygdala afferents to the mPFC projectome (Papilloud et al., 2019, 2020). These studies also show that manipulating GR with anti‐glucocorticoids presents an option to *reset* a stress‐coping and adaptation mechanism of which recently more details have been reported (Dalm et al., 2019). While MR plays a role in risk assessment and selection of coping style during coping with escapable and controllable stressors, such a role of the high affinity MR is not yet systematically studied during coping with an inescapable stressor. GR activation promotes contextual memory consolidation irrespective the extent of control (Figure 2).

## 9 CONCLUDING REMARKS

From our poll results we can conclude that around 2015 a plateau is reached in the number of FST publications with contributions from notably China and South‐America proportionally increasing over time. Stress effects are relatively more examined in rats, while the mouse is favourite in antidepressant screening assays. Eighty percent of the studies are performed with male animals and from the relatively few studies it can be learned that female animals display higher immobility at retest which can be reduced by antidepressant treatment (Kokras et al., 2015). In fact, female animals are understudied in coping with inescapable stressors.

In response to our query the Editors‐in‐Chief responded that they rely on the expertise of their reviewers and rather not interfere. If asked about their own opinion most Editors acknowledged that the FST is not a model of depression or offered no personal opinion. The Editors of *Psychoneuroendocrinology* recently adopted the policy to draw attention of the authors to the NIMH considerations regarding the use of animal neurobehavioural approaches in basic and preclinical studies, while reminding them ‐without being prescriptive‐ of alternative explanations to FST immobility (see: https://grants.nih.gov/grants/guide/notice‐files/NOT‐MH‐19‐053.html).

To that effect the relevant paragraph in the NIMH notice reads “The NIMH recommends the use of models “for” addressing neurobiological questions rather than models “of” specific mental illnesses. Similarly, NIMH strongly discourages description of animal behaviors in terms of emotions and thought processes that are accessible only in humans by self‐report (e.g., terms such as depressed, anxious, lonely) or through clinical diagnoses.” This statement may of course invite cosmetic solutions for describing the actual experiment, but its “essence” is in the importance of the hypothesis, question and mechanism (see Box 2). Interestingly, in the 1980s we felt it better to simply state “acquired immobility” or even more neutral “retention of a behaviour” (de Kloet et al., 1988; Veldhuis et al., 1985) to describe what is actually observed. Today coping in the FST is considered the default survival mode which leaves a molecular signature in an engram of sparsely distributed hippocampal dentate gyrus neurons under control of GR activation (de Kloet et al., 1988; Nasca et al., 2015; Gutierrez‐Mecinas et al., 2011; Kaouane et al., 2012; Rao‐Ruiz et al., 2019; Reul et al., 2015; Revest et al., 2014).

**Outstanding research questions**

1. How do bottom‐up endocrine and top‐down mPFC circuitry interact in management of stress‐coping?
2. Where and how is the functional specialization organized in mPFC neuronal ensembles during selection of stress‐coping styles?
3. How are MR‐ and GR‐mediated actions coordinated during coping with escapable or inescapable stressors?
4. What are the sex differences in stress‐coping with inescapable stressors? This question is with respect to females and the reproductive cycle understudied (Kokras et al., 2015).
5. What is the appropriate behavioral *z*‐score for assessment of stress‐coping ability and resilience? See also the web‐based RePAIR application for reduction of animal use. (https://vbonapersona.shinyapps.io/repair_app_submit/; Bonapersona, Kentrop, et al., 2019).

The NIHM notice is also relevant for a severity analysis to achieve further insight in the three R’s in animal welfare: Replacement, Reduction, and Refinement (Reardon, 2019), when the generated FST database can be made available to optimize protocols. How this occurs can be learned from a recently accomplished meta‐analysis of early life adversity outcome. It produced a user friendly MaBapp (https://osf.io/ra947/), which is accessible for researchers to run tailor‐made meta‐analyses, for the choice of optimal experimental protocols and power calculation of the investigation (Bonapersona, Kentrop, et al., 2019). More recently, Valeria Bonapersona developed RePAIR, a web‐based tool that enabled retrieval of control data from previous animal experiments to improve the power in statistical analysis. The application relies on the observation that control groups generally are similar. In a simulation analysis power increase up to 100% with half the required sample size (Bonapersona, Hoijtink, et al., 2019). Then, on another level timely machine learning methods will be increasingly applied in animal research for instance for, for example, recognition and classification of facial expressions that may reflect emotional states (Dolensek et al., 2020).

An integrated computation of *z*‐normalization across behavioural tests is an option to stratify outcome measures (see Box S3). Such a *z*‐score would allow standardization of the measurement as an approach to increase the reliability of the behavioural phenotyping (Guilloux et al., 2011; Weger et al., 2020). Order effects are likely to be relevant for calculating these *z*‐scores, given that exposure to one test could have lasting effects on behaviour in a following test. We found that the sequence of test exposure (e.g., SPT and FST or OFT and FST) was most often such that the FST was applied as the final test. Some authors justify the use of this typical order with the notion that the most aversive test should be applied as last test because it could affect behaviour in subsequent tests (Perrot‐Sinal et al., 2004). However, we found very little data justifying this assumption. In fact, data exist showing opposite trends, so that behaviour in the FST was most affected by order of testing, in an experimental sequence including the zero maze and the OFT (Blokland et al., 2012). In our opinion, *z*‐scores need to take into account the type of behavioural paradigm related to the nature of the stressor (escapable or inescapable, controllable or uncontrollable, psychological or physical), since we are only beginning to understand the different neuronal substrates for these conditions.

Addressing the right neurobiological question is fundamental in the RDOC strategy (see: https://www.nimh.nih.gov/research‐priorities/rdoc/definitions‐of‐the‐rdoc‐domains‐andconstructs.shtml#part_154187) launched more than 10 years ago. The strategy aims for a charting over various levels of biological complexity from gene to behaviour called units of analysis. The outcome of this systematic analysis hopefully will facilitate to identify critical mechanisms in the *translation of psychological constructs to domains of human behaviour*.

In our previous article (Molendijk & de Kloet, 2019) we have highlighted a quote from the late Bruce McEwen on page 6 of the inaugural issue of the journal Chronic Stress: “cortisol acts in 6 RDoc units of analyses from gene to behavior; the hormone can alter arousal and regulatory circuitry and affects psychosocial, cognitive, positive‐ and negative valence systems. Accordingly, it seems therefore that the RDoc framework does not yet fully recognize the role of stress and stress hormones in coordinating the domains over the various units of analysis in neuroendocrine, immune and metabolic interactions.” (McEwen, 2017).

Indeed, cortisol and corticosterone coordinate circadian events and the organism's response to environmental, physical and psychogenic stressors. This action exerted by the steroids is mediated in complementary fashion by MR and GR on the genomic and non‐genomic level (de Kloet et al., 2005, 2018). MR and GR are expressed in the mPFC circuitry. The hippocampal, amygdala and VTA dopaminergic inputs are richly endowed with both receptors and modulate upon activation emotional, contextual, motivational and valuation inputs to executive control (Douma & de Kloet, 2020). Chronic stress affects integrity and recruitment of the neuronal ensembles in the mPFC projections as well as in its limbic inputs (McEwen, 2017; McKlveen et al., 2019). How glucocorticoids are implicated is a challenge for future research (see Figures 2 and 3; Box S4).

Much progress has been made in unravelling the function of the mPFC projectome with its avBNST hub in control of vl‐PAG motor output conveying motor inactivity and PVN‐mediated adrenal glucocorticoid secretion with its contextual, emotional and valence inputs (McKlveen et al., 2019; Radley & Johnson, 2018; Sandi & Haller, 2015). Alternatively, GR responsive genes contribute significantly to an antidepressant responsive gene networks in mouse and man supporting further a key role of glucocorticoids in resilience (Carrillo‐Roa et al., 2017). It will be a challenge to unravel how the *bottom‐up* endocrine and *top‐down* mPFC circuitry interact in management of stress‐coping, adaptation and resilience during acute and chronic stress conditions. The avenues of research towards this goal are sketched in a recent book on “Stress Resilience” (Chen, 2019) which contains also contributions of Bruce McEwen and of Carmen Sandi and Mathias Schmidt, the editors of this Special Issue of the European Journal of Neuroscience. To see the results of these forthcoming investigations will be an exciting prospect.

## CONFLICT OF INTEREST

Marc Molendijk has no potential competing (financial) interests to report. Ron de Kloet is on the scientific advisory board of Dynacorts Therapeutics, and owns stock of Corcept Therapeutics.

### Peer Review

The peer review history for this article is available at https://publons.com/publon/10.1111/ejn.15139.

## Supporting information

Supplementary Material

## Data Availability

The data used in this article are available upon reasonable request.
