# Supplementary material for: Forced swim stressor: Trends in usage and mechanistic consideration
Source: Eur J Neurosci. 2021 Mar 8;55(9-10):2813–31. doi: 10.1111/ejn.15139 (PMC9291081; doi:10.1111/ejn.15139)
Supplement: Supplementary file 1 — Supplementary Material [file EJN-55-2813-s001.docx]

**SUPPLEMENTARY MATERIALS**

**Table S1**. Frequency of conjunct use of the FST with either the TST, SPT, EPM, LDB, and OFT.

|  | *n* (*N*) | *n* (%) TST, SP | *n* (%) EPM, LDB, OFT | *n* (%) LoMoAc ^2^ |
| --- | --- | --- | --- | --- |
| *Overall* | 261 (1,302) | 107 (41%) | 139 (55%) | 193 (74%) |
| *Behavioral Brain Research* | 71 (355) | 27 (38%) | 47 (61%) | 51 (72%) |
| *Biological Psychiatry* | 47 (235) | 31 (66%) | 30 (55%) | 37 (79%) |
| *Neuroscience* | 56 (289) | 19 (34%) | 32 (52%) | 45 (80%) |
| *Physiology & Behavior* | 47 (235) | 19 (40%) | 29 (58%) | 39 (83%) |
| *Psychoneuroendocrinology* | 40 (200) | 12 (30%) | 19 (48%) | 20 (50%) |

*^1^* *N* = estimated total number of published papers in the journal in the row in the past 10 years. *n* = number of papers that we randomly selected for the purposes of our study.

*^2^* LoMoAc; locomotor activity

**Table S2**. Interpretation of immobility

|  | *n* | DL behavior *n* (%) | AD response *n* (%) | Coping *n* (%) | *Other n* (%) |
| --- | --- | --- | --- | --- | --- |
| *Overall* | 261 | 171 (66%) | 39 (15%) | 11 (4%) | 40 (15%) |
| *Behavioral Brain Research* | 71 | 50 (70%) | 12 (17%) | 2 (3%) | 7 (9%) |
| *Biological Psychiatry* | 47 | 27 (57%) | 15 (32%) | 2 (2%) | 4 (8%) |
| *Neuroscience* | 56 | 36 (64%) | 8 (14%) | 0 (0%) | 12 (22%) |
| *Physiology & Behavior* | 47 | 34 (72) | 2 (4%) | 2 (4%) | 9 (19%) |
| *Psychoneuroendocrinology* | 40 | 24 (60) | 2 (5%) | 6 (15%) | 8 (20%) |

| **BOX S1.** Short description of the TST the SPT, and the anxiety read-outs EPM, LDB, and OFT. |
| --- |
| *The Tail Suspension Test – TST*  In this test, mice are suspended by the tail for 6 minutes. As in the FST, first the animal tries to escape from this situation by active behavior (*e.g.,* struggling) but after some time the animal will resume an immobile posture. Immobility is the outcome measure of this experiment (Castagné et al., 2011). The TST was presented in the eighties as a test that is sensitive to screen for antidepressant potential (Steru et al., 1985), but it has become common ground to use it as a model for despair or depression (Castagné et al., 2011). .  *The Sucrose Preference Test – SPT*  The SPT is a reward-based test. The SPT apparatus consists of a two-bottle choice paradigm. One of the bottles contains a sweet solution, the other plain water. A reduction of preference ratio for the sweet solution relative to the non-sweet solution is said to reflect anhedonia, that is the inability to experience pleasure. The preference for the sweet solution is sensitive for antidepressant drugs (Liu *et al.,* 2011).  *The Elevated Plus Maze – EPM*  Probably for reasons of safety, rodents have the tendency to remain in enclosed spaces but they also have the tendency to explore. The EPM is based on these conflicting tendencies. The test situation consists of a maze with 2 open and 2 closed arms. The proportion of time that the animal spends in the closed arms of the maze is used as an indication for the anxiety levels of the animal (Pellow *et al.,* 1985). Some types of anxiolytics reduce the time that an animal spends in the closed arms (Sarkar 2020).  *The Light-Dark Box -- LDB*  Rodents in general prefer dark compartments over compartments that are brightly lit. As in the EPM, this likely is due to safety. The LDB is an apparatus that is composed out of a larger dark component and a smaller light compartment. It is inferred from this test that in case the animal spends relatively more time in the dark component the animal is relatively anxious (Crawley and Goodwin, 1980). Some types of anxiolytic drugs increase the time spent in the brightly lit compartment (Sarkar 2020).  *The Open Field Test -- OFT*  Just as the EPM and the LDB, the OFT is based on the tendencies of rodents to seek safety and to explore. In the OFT, the animal is placed in an arena that is enclosed by walls. Locomotion and position of the animal during a certain interval are assessed. Little locomotion and a preference to stay close to the walls of the arena are used as an indication for anxiety. (Hall 1936; Sarkar 2020). This behavior is also sensitive to anxiolytic drugs (Sarkar 2020). |

| **BOX S2.** Trends in the use of the Forced Swim Test |
| --- |
| *The two-stage FST experiment; induce immobility 🡪 reduce immobility*  During the past years, however, more-and-more researchers apply a two-stage FST approach. Immobility is targeted first by stress exposure (*e.g.,* maternal separation or social defeat stress) and this is the starting point in the second stage of the experiment. In this stage the induced immobility is normalized by a pharmacological or non-pharmacological manipulation. Papadakakis et al. (2019) provide an example for this. They submitted newborn rats to maternal separation for 3h daily (11.00-14.00h) from postnatal day (pnd) 2-14 and subsequently rats were exposed from pnd 21-76 for 12h per day during their active time to Mozart’s sonata K. 448 vs ambient noise. The rats separated from the dam showed decreased sociability, spend more time in the closed arm of the elevated plus maze and displayed increased FST immobility times at retest, while the number of dendritic spines of hippocampal CA1 neurons was reduced. Mozart’s sonata attenuated these effects towards control levels leading the authors to conclude that musical enrichment has an anti-depressant effect in rats. We estimate that currently in about 10 percent of the FST papers such a two-stage approach is used.  Effects of diet and microbiome variation on stress-induced FST behavior  The current interest in the effect of dietary components on mood (Thomas-Odenthal et al., 2020) left its mark on the use of the FST. In fact, a quarter of the current papers that use the FST do so to test the effects of dietary components or herbs or gut bacteria, whereas a decade ago this number was negligible. Often, so called unhealthy food items are investigated for their depressogenic properties. An example is the experiment by Estrela Costa et al. (2015) in which rats were exposed to a cafeteria-like diet (incl. cola-like soft drinks and potato chips). The authors observed a higher amount of immobility in the - by then obese - rats that were exposed to stress and the cafeteria diet. The immobility was interpreted as a depression-like behavior of the fat rats, induced by a poor-quality diet, although alternatively body fat usually facilitates floating.  Effect of ketamine on stress-induced FST behavior  Fewer-and-fewer FST articles are published that test the effects of pharmacological agents on immobility time, with the exception of ketamine. We estimate that of all the pharmacological studies that were published in the past 5 years, 75% are studies on ketamine or norketamine, whereas this number was about zero 10 years ago. Ketamine reduces immobility time. An example is given by Khakpai et al. (2019). They showed that an intra-peritoneal injection of ketamine reduced immobility time in the 1-day FST variant, notably when it was administered with cannabinoid receptor antagonists. The conclusion was that the interaction between ketamine and the cannabinoid system may modulate depression related behavior. Ketamine, however, also induces locomotor activity (Imre et al., 2006) which can explain observed differences in time spent immobile. Ketamine also impairs memory in rodents and humans (Imre et al., 2006; Pérez et al., 2019), and this is relevant for understanding the immobility response, notably when the 2-day FST variant is used. |
| **BOX S3.** Integrated behavioral z-scoring |
| Behavioral z-scoring refers to a method to phenotype behaviour over several complementary paradigms. The first application of this method was described by Guilloux et al. in 2011. They exposed mice to a chronic mild stress paradigm or to the stress hormone corticosterone for a prolonged period of time with the aim to induce *emotion like states*. The animals were tested in the elevated plus maze, open field test, and novelty suppressed feeding test, under the assumption that these tests all tap different aspects of ‘emotionality’ on part of the animal. The data over tests was z-normalized and overall ‘emotionality’ scores were calculated by summing over single scores. The authors found that the integrated ‘emotionality’ z-score could be altered by stress exposure and by antidepressant treatment. The composite z-score has lower variance relative to single test scores and as such it comes with the advantage of a higher level of statistical power (Guilloux et al. 2011). Another advantage is that since the score is a composite, its reliability can be tested for. In addition, the integrated ‘emotionality’ z-score reduces the likelihood of false positive findings given that fewer statistical tests are performed (Labots et al. 2018).  Integrated behavioral z-scoring has been presented being similar to the symptom-based diagnosis that is in use in psychiatry and clinical psychology (Guilloux et al. 2011), but it is not a solution for validity issues of pre-clinical measures or tests. For instance, an integrated z-score calculated over the FST and TST probably will provide a more reliable and precise estimate about the construct that underlies the response to these tests (see **Box S1** and **Table 1** for conceptual FST-TST overlap and similarity in response type over experiments) relative to the score on either one of these tests. However, integrated behavioral z-scoring is no license for using unvalidated measures. |

| **BOX S4.** Mineralocorticoid (MR) and Glucocorticoid receptors (GR) |
| --- |
| The naturally occuring glucocorticoids, cortisol and corticosterone, bind with a tenfold higher affinity to  MR than to GR, which are nuclear receptors that regulate gene transcription as homo- and heterodimers.  The hormones also exert non-genomic actions via MR and GR. Non-genomic and genomic MR activation  promote excitatory transmission, which is suppressed via GR. MR is abundantly expressed in hippocampus,  amygdala, lateral septum, but occur in lower concentrations also in mPFC neurons and elsewhere in the  forebrain. The enzyme (11β-hydroxysteroid dehydrogenase type 2) converts cortisol and corticosterone to  their bio-inactive congeners and is in brain abundantly expressed in a discrete number of neurons in the  n.tractus solitarii (NTS) and confers aldosterone-specificity to colocalized MR with a role in salt appetite and  cardiovascular regulation. GR is co-localized with MR and widely distributed with highest density in stress  regulating centers such as the paraventricular nucleus, the limbic-prefrontal cortical regions and the  ascending aminergic neurons. The role of GR is to provide energy substrates for tissues in need and to  protect body and brain to its own initial defense reactions. MR is involved in initiation of the stress response  which is terminated via GR. MR- and GR-mediated signaling act in a complementary manner and needs to be  in balance for maintenance of homeostasis and health. See for reviews on the scien of stress, glucocorticoids  and the brain (de Kloet et al., 2018) (Gasparini et al., 2018) (Häusl et al., 2019) (Herman and Tasker, 2016)  (Joëls et al., 2012) (McEwen et al., 2015)(Sandi and Haller, 2015) (Sapolsky et al., 2000) |

**References**

Castagné, V., Moser, P., Roux, S., & Porsolt, R. D. (2011). Rodent models of depression: forced swim and tail suspension behavioral despair tests in rats and mice. *Current Protocols in Neuroscience*, *55*(1), 8-10. <https://doi.org/10.1002/0471142301.ns0810as55>

Crawley, J., & Goodwin, F. K. (1980). Preliminary report of a simple animal behavior model for the anxiolytic effects of benzodiazepines. *Pharmacology Biochemistry and Behavior*, *13*(2), 167-170. <https://doi.org/10.1016/0091-3057(80)90067-2>

da Costa Estrela, D., da Silva, W. A. M., Guimarães, A. T. B., de Oliveira Mendes, B., da Silva Castro, A. L., da Silva Torres, I. L., & Malafaia, G. (2015). Predictive behaviors for anxiety and depression in female Wistar rats subjected to cafeteria diet and stress. *Physiology & Behavior*, *151*, 252-263. <https://doi.org/10.1016/j.physbeh.2015.07.016>

de Kloet, E.R., Meijer, O.C., de Nicola, A.F., de Rijk, R.H., Joëls, M., 2018. Importance of the brain corticosteroid

receptor balance in metaplasticity, cognitive performance and neuro-inflammation. *Front. Neuroendocrinol*.

49, 124–145. https://doi.org/10.1016/j.yfrne.2018.02.003

Gasparini, S., Resch, J.M., Narayan, S. V., Peltekian, L., Iverson, G.N., Karthik, S., Geerling, J.C., 2018.

Aldosterone-sensitive HSD2 neurons in mice. *Brain Struct. Funct*. https://doi.org/10.1007/s00429-018-1778-y

Guilloux, J. P., Seney, M., Edgar, N., & Sibille, E. (2011). Integrated behavioral z-scoring increases the sensitivity and reliability of behavioral phenotyping in mice: relevance to emotionality and sex. *Journal of Neuroscience methods*, *197*(1), 21-31. <https://doi.org/10.1016/j.jneumeth.2011.01.019>

Hall, C. S. (1936). Emotional behavior in the rat. III. The relationship between emotionality and ambulatory activity. *Journal of Comparative Psychology*, *22*(3), 345.

Häusl, A.S., Balsevich, G., Gassen, N.C., Schmidt, M. V., 2019. Focus on FKBP51: A molecular link between

stress and metabolic disorders. *Mol. Metab.* 29, 170–181. https://doi.org/10.1016/j.molmet.2019.09.003

Herman, J.P., Tasker, J.G., 2016. Paraventricular hypothalamic mechanisms of chronic stress adaptation. *Front.*

*Endocrinol*. (Lausanne). https://doi.org/10.3389/fendo.2016.00137

Imre, G., Fokkema, D. S., Den Boer, J. A., & Ter Horst, G. J. (2006). Dose–response characteristics of ketamine effect on locomotion, cognitive function and central neuronal activity. *Brain Research Bulletin*, *69*(3), 338-345. <https://doi.org/10.1016/j.brainresbull.2006.01.010>

Joëls, M., Sarabdjitsingh, R.A., Karst, H., 2012. Unraveling the time domains of corticosteroid hormone

influences on brain activity: rapid, slow, and chronic modes. Pharmacol. Rev. 64, 901–38.

https://doi.org/10.1124/pr.112.005892

Khakpai, F., Ebrahimi-Ghiri, M., Alijanpour, S., & Zarrindast, M. R. (2019). Ketamine-induced antidepressant like effects in mice: A possible involvement of cannabinoid system. *Biomedicine & Pharmacotherapy*, *112*, 108717. <https://doi.org/10.1016/j.biopha.2019.108717>

Labots, M. M., Laarakker, M. M., Schetters, D. D., Arndt, S. S., & van Lith, H. H. (2018). An improved procedure for integrated behavioral z-scoring illustrated with modified Hole Board behavior of male inbred laboratory mice. *Journal of Neuroscience Methods*, *293*, 375-388. <https://doi.org/10.1016/j.jneumeth.2017.09.003>

Liu, M. Y., Yin, C. Y., Zhu, L. J., Zhu, X. H., Xu, C., Luo, C. X., ... & Zhou, Q. G. (2018). Sucrose preference test for measurement of stress-induced anhedonia in mice. *Nature Protocols*, *13*(7), 1686-1698. <https://doi.org/10.1038/s41596-018-0011-z>

McEwen, B.S., Bowles, N.P., Gray, J.D., Hill, M.N., Hunter, R.G., Karatsoreos, I.N., Nasca, C., 2015. Mechanisms

of stress in the brain. *Nat. Neurosci*. 18, 1353–1363. https://doi.org/10.1038/nn.4086

Papadakakis, A., Sidiropoulou, K., & Panagis, G. (2019). Music exposure attenuates anxiety-and depression-like behaviors and increases hippocampal spine density in male rats. *Behavioural Brain Research*, *372*, 112023. <https://doi.org/10.1016/j.biopha.2019.108717>

Pellow, S., Chopin, P., File, S. E., & Briley, M. (1985). Validation of open: closed arm entries in an elevated plus-maze as a measure of anxiety in the rat. *Journal of Neuroscience Methods*, *14*(3), 149-167. <https://doi.org/10.1016/0165-0270(85)90031-7>

Pérez, M. Á., Morales, C., Santander, O. C., García, F., Gómez, I., Peñaloza-Sancho, V., ... & Fuenzalida, M. (2019). Ketamine-treatment during late adolescence impairs inhibitory synaptic transmission in the prefrontal cortex and working memory in adult rats. *Frontiers in Cellular Neuroscience*, *13*, 372. <https://doi.org/10.3389/fncel.2019.00372>

Sandi, C., Haller, J., 2015. Stress and the social brain: behavioural effects and neurobiological mechanisms. *Nat.*

*Rev. Neurosci.* 16, 290–304. https://doi.org/10.1038/nrn3918

Sapolsky, R.M., Romero, L.M., Munck, A.U., 2000. How do glucocorticoids influence stress responses?

Integrating permissive, suppressive, stimulatory, and preparative actions. *Endocr. Rev*. 21, 55–89.

https://doi.org/10.1210/er.21.1.55

Sarkar, D. (2020). A review of behavioral tests to evaluate different types of anxiety and anti-anxiety effects. *Clinical Psychopharmacology and Neuroscience*, *18*(3), 341-351. <https://orcid.org/0000-0003-3165-6857>

Steru, L., Chermat, R., Thierry, B., & Simon, P. (1985). The tail suspension test: a new method for screening antidepressants in mice. *Psychopharmacology*, *85*(3), 367-370.  [https://doi.org/10.1007/BF00428203](https://psycnet.apa.org/doi/10.1007/BF00428203)

Thomas-Odenthal, F., Molero, P., van der Does, W., & Molendijk, M. (2020). Impact of review method on the conclusions of clinical reviews: A systematic review on dietary interventions in depression as a case in point. *PloS One*, *15*(9), e0238131. <https://doi.org/10.1371/journal.pone.0238131>
